# Guillain-Barré syndrome and COVID-19 vaccines: focus on adenoviral vectors

**DOI:** 10.3389/fimmu.2023.1183258

**Published:** 2023-04-26

**Authors:** Piotr Rzymski

**Affiliations:** Department of Environmental Medicine, Poznan University of Medical Sciences, Poznan, Poland

**Keywords:** Guillain - Barré, SARS-CoV-2, pandemic, Adenoviral (Ad) vector, COVID-19 vaccination

## Abstract

COVID-19 vaccination is a life-saving intervention. However, it does not come up without a risk of rare adverse events, which frequency varies between vaccines developed using different technological platforms. The increased risk of Guillain-Barré syndrome (GBS) has been reported for selected adenoviral vector vaccines but not for other vaccine types, including more widely used mRNA preparations. Therefore, it is unlikely that GBS results from the cross-reactivity of antibodies against the SARS-CoV-2 spike protein generated after the COVID-19 vaccination. This paper outlines two hypotheses according to which increased risk of GBS following adenoviral vaccination is due to (1) generation of anti-vector antibodies that may cross-react with proteins involved in biological processes related to myelin and axons, or (2) neuroinvasion of selected adenovirus vectors to the peripheral nervous system, infection of neurons and subsequent inflammation and neuropathies. The rationale behind these hypotheses is outlined, advocating further epidemiological and experimental research to verify them. This is particularly important given the ongoing interest in using adenoviruses in developing vaccines against various infectious diseases and cancer immunotherapeutics.

## 1 Introduction

The COVID-19 pandemic has been met with unprecedented scientific efforts, including developing and clinical testing numerous vaccine candidates at an unseen pace (1, 2). China reported the first cluster of cases in late December 2019, the first genomic data of its etiological agent, later named severe acute respiratory syndrome coronavirus 2 (SARS-CoV-2), became publicly available in the first half of January 2020 (3), while the first COVID-19 vaccines were authorized by the end of 2020. By the end of 2021, over twenty different COVID-19 vaccines were already in use in various world regions (4). These included preparations based on the inactivated virus (e.g., BIBP-CorV by Sinopharm and CoronaVac by Sinovac Biotech), mRNA technology (e.g., BNT162b2 by BioNTech/Pfizer, and mRNA-1273 by Moderna), non-replicating adenoviral vector (e.g., AZD1222 by AstraZeneca, Ad26.COV2.S by Janssen/Johnson&Johnson, Ad5-nCoV by Cansino Biologics, and Sputnik V by Gamaleya Research Institute of Epidemiology and Microbiology), plasmid DNA (ZyCoV-D by Cadila Healthcare), and subunit protein (e.g., NVX-CoV2373 by Novavax) (4).

The COVID-19 vaccination has been proven to be a life-saving intervention. As estimated, it reduced COVID-19 mortality by two-thirds in 2021, averting globally 19.8 million deaths (5). It was also associated with lower admissions rates to intensive care units and hospitalizations (6–8), and evidence shows that it also reduced long-term consequences of SARS-CoV-2 infections, known as post-COVID syndrome (6, 7). With time it became evident that booster doses are required to restore gradually decreasing levels of neutralizing antibodies and address the novel SARS-CoV-2 variants that revealed varying levels of immune evasion (8, 9). Moreover, in response to the emergence of the Omicron variant and its further evolution, the bivalent mRNA vaccines, containing the second component optimized for Omicron BA.1 or BA.4/BA.5 subvariants, were authorized in the second half of 2022. All in all, this resulted in repeated administration of COVID-19 vaccines, with over 13 billion doses administrated by the end of 2022 and 69% of the world population receiving at least one dose (10).

Although the clinical trials have demonstrated a good safety profile of COVID-19 vaccines, with the reactogenicity represented mainly by short-term local (e.g., injection-site pain, redness, swelling) or systemic responses (e.g., fatigue, fever, headache), one should note that these studies, despite thousands of participants, were not designed to detect very rare adverse events that may occur during the massive vaccination campaign. For example, after authorization, mRNA vaccination has been associated with an increased risk of myocarditis and pericarditis (11), while unusual thrombotic events associated with thrombocytopenia have been recorded following the administration of adenoviral vector vaccines (12, 13). Moreover, some adenoviral vaccines were also associated with the risk of Guillain-Barré syndrome (GBS), a rare, immune-mediated neurologic disorder characterized by varying degrees of weakness, sensory abnormalities, and autonomic dysfunction due to damage to peripheral nerves and nerve roots (14). GBS is usually reversible, but in severe cases, it may lead to breathing difficulties, require mechanical ventilation, and leave permanent neurological alterations (15). Its worldwide annual incidence is estimated at 0.8-1.9 cases per 100,000 persons, with frequency increasing with age and being more common in men (16).

In 2021, the population-based study employing data from the National Health Service in England identified that administration of the first dose of AZD1222, based on a modified chimpanzee adenovirus (ChAdOx1), was associated with an excess of GBS risk of 0.58 cases per 100,000 doses (17). In India, the reported frequency of GBS following AZD1222 was 1.4- to 10-fold higher than expected for a population and included severe cases progressing to areflexic quadriplegia (18), while in Australia, it exceeded the background level by 4.7-fold (19). In 2021, a preliminary safety concern for GBS following a single dose of Ad26.COV2.S vaccination, based on modified human adenovirus serotype 26 (HAdV26), was detected in the United States, with the observed-to-expected ratio of 4.2 over the 42-day observation (20). More recently, this concern was confirmed by the analysis of the U.S. Vaccine Adverse Event Reporting System (21). As observed, the administration of Ad26.COV2.S vaccine was associated with an increased risk of GBS, with the observed-to-expected ratio of 3.8 and 2.3 within 21 and 42 days of vaccination, respectively (21). Analysis of VigiBase, which contains spontaneous reports of adverse drug reactions from 149 countries, again indicated that adenoviral vector COVID-19 vaccines AZD1222 and Ad26.COV2.S are associated with the increased observed-to-expected ratio of GBS consistently exceeding 2.0 for all analyzed countries (22). Cases of GBS were also reported following the administration of the first dose of the Sputnik V adenoviral vaccine (containing HAdV26), and its modification Sputnik Light (that is also based on the same adenoviral vector), was also reported in the literature (23, 24). However, no comprehensive epidemiological surveillance data for these vaccines encompassing GBS was published. Interestingly though, no increased risk of GBS was seen in the case of Ad5-nCoV (although GBS cases after its administration were reported) (25, 26), which is the only adenoviral COVID-19 vaccine based solely on human adenovirus serotype 5 (HAdV5) given as a single dose (27).

Although the GBS risk is increased following the administration of certain adenoviral COVID-19 vaccines, cases of this condition remain rare. This indicates the potential existence of a susceptible group of individuals among vaccinees. Such a susceptibility may be due to genetic predisposition linked, albeit not without controversies, to genes encoding human leukocyte antigens, a cluster of differentiation 1A, FAS, Fc gamma receptors, intercellular adhesion molecule-1, nucleotide oligomerization domain, toll-like receptor 4, tumor necrosis factor-α, and various interleukins (28). However, the association between COVID-19 vaccination and polygenic GBS risk was not subject to any study that the author of this paper is aware of. Nevertheless, such genetic predisposition cannot solely explain why GBS cases following COVID-19 vaccination are biased toward some of the adenoviral vector vaccines. Examining the mechanisms behind this phenomenon is pivotal in taking precautionary measures, adjusting recommendations, and managing vaccine hesitancy. Similarly to the need to understand whether an increased risk of peri- and myocarditis is associated solely with COVID-19 mRNA vaccines or mRNA technology in general (29, 30), it is essential to elucidate the exact nature of the relationship between GBS and adenoviral vaccines. Such knowledge can be potentially beneficial in improving the technological platforms employing adenoviruses as gene delivery systems. It is also imperative given that non-replicating and single-cycle adenoviruses have been recognized as suitable vaccine vectors as they can be easily modified and manufactured, revealing a broad spectrum of host cell tropism and high gene expression, and, consequently, inducing a robust innate and adaptive response (31). Currently, adenoviruses are utilized in authorized vaccines against COVID-19 (**Table 1**) and filoviruses (Ad26.ZEBOV by Janssen/Johnson&Johnson) (32). No safety signals regarding GBS were observed during clinical trials of Ad26.ZEBOV vaccine (based on adenovirus type 26, similarly to Ad26.COV2.S) (33–36), although one should note that such studies are not designed to detect very rare adverse events, while at the same time, contrary to Ad26.COV2.S, there is no broad post-authorization experience with this vaccine.

**Table 1:** Authorized COVID-19 vaccines using adenoviral vectors.

Name	Manufacturer	Doses (primary regime)	Vector	Encoded spike protein	GBS risk
AZD1222	Oxford/AstraZeneca	2	ChAdOx1	Wild-type	Increased
Ad26.COV2.S	Janssen/Johnson&Johnson	1	HAdV26	Stabilized prefusion	Increased
Ad5-nCoV	Cansino Biologics	1	HAdV5	Wild-type	Not increased
Sputnik V	Gamaleya Research Institute	2	Heterologous. First dose: HAdV26 Second dose: HAdV5	Wild-type	Not enough data
Sputnik Light		1	HAdV26	Wild-type	

ChAdOx1, adenoviral vector based on chimpanzee adenovirus Y25; GBS, Guillain-Barré syndrome; HAdV26, human adenovirus serotype 26; HAdV5, human adenovirus serotype 5.

The clinical trials of various adenoviral vector vaccine candidates are ongoing, including preparations against other coronaviruses (MERS-CoV), influenza, malaria, RSV, tuberculosis, and Chikungunya virus, and even more candidates are in earlier stages of testing (37–40). In addition to classical intramuscular injections, adenoviral vaccine candidates for oral, intradermal, and intranasal administration have also been developed. Such routes are substantially less invasive and painless, hence more attractive for individuals with needle phobia, while potentially offering additional immunological benefits (e.g., sterilizing immunity in the case of intranasal preparations) (41–43). There is also an interest in exploring adenoviral-based cancer gene therapies (44, 45). In other words, a portfolio of medical use of adenoviruses is likely to increase in the future; thus, understanding all potential safety issues of their administration is highly important.

## 2 The hypotheses

This paper hypothesizes that the administration of selected adenoviruses employed in the vector vaccines is triggering the adaptive response to the viral vector that can eventually lead to autoimmunity against proteins of the peripheral nervous system and induce GBS. An alternative hypothesis is also presented and implies the neuroinvasion of selected adenoviral vectors, their interaction with peripheral neurons, and the induction of subsequent inflammation and neuropathies associated with GBS.

## 3 Evaluating the hypotheses

### 3.1 Guillain-Barré syndrome and spike protein

In general, the etiology of GBS remains not fully known. It is suggested to be predominantly an autoimmune condition resulting from molecular mimicry. Under this scenario, autoantibodies, induced by an external factor, target epitopes on peripheral nerves and induce axonal damage and neuronal demyelination (46, 47). Therefore, some authors suggested that GBS following the administration of the COVID-19 adenoviral vector vaccine is due to cross-reaction between generated antibodies against SARS-CoV-2 spike protein and neuronal proteins (48–50). Moreover, using a bioinformatic approach the molecular mimicry was predicted between spike protein epitopes and sequences of eight proteins involved in biological processes related to myelin and axons, i.e., neural cell adhesion molecule, receptor-type tyrosine-protein phosphatase zeta, teneurin-4, receptor tyrosine-protein kinase erbB-2-, integrin alpha-X, integrin beta-1, attractin, and myelin-associated glycoprotein (51). Therefore, the cross-reactivity of anti-spike antibodies with these proteins could potentially lead to severe neurological conditions, such as GBS.

However, if this assumption holds, GBS should be frequently observed in COVID-19 patients and individuals vaccinated with any COVID-19 vaccine since they all use spike protein as an antigen. A systematic review of 79 papers published until February 2021 identified 109 GBS cases related to COVID-19, including 16 cases in the United States (52). However, by the time this review was performed, over 103 million cases of SARS-CoV-2 had been confirmed worldwide, of which nearly 26.5 million were reported in the United States (10). It indicates that the frequency of GBS in COVID-19 is much lower than expected when assuming the involvement of anti-spike antibodies, especially considering that most infected patients produce their detectable levels (53–55). Moreover, some COVID-19 patients who presented with GBS tested positive for antibodies against antigangliosides (52), which can be implicated in selected variants of this condition (56). Furthermore, the reported GBS cases were more common in men, which aligns with the general tendency observed for this condition (16). Therefore, it can be suggested that GBS is a rare complication in COVID-19, which pathogenesis is unlikely due to cross-reactive anti-spike antibodies, but possibly result from other causes, e.g., neuroinvasion of SARS-CoV-2 (as there are reports on its presence in cerebrospinal fluid and brain tissue) (57). It may also be due to particular genetic predispositions postulated to influence the GBS risk (28).

Moreover, although GBS cases were observed in individuals vaccinated with different COVID-19 vaccines, its increased risk was only found for adenoviral vector vaccines (19, 21, 22, 58). Therefore, European Medicine Agency included a warning of GBS in the updated versions of package information of AZD1222 and Ad26.COV2.S vaccines (59, 60), but not for other COVID-19 vaccines. In many developed regions, e.g., the United States and European Union, the vaccination campaigns were disproportionally based on mRNA vaccines (e.g., nearly 1 billion doses of mRNA vaccines vs. approximately 185 million doses of adenoviral vector vaccines were given in the European Economic Area by early February 2023) (61). Therefore, if the post-vaccination GBS was related to cross-reactive anti-spike antibodies generated after immunization, it should be predominantly observed after mRNA, not adenoviral vaccination, but the evidence points to the contrary (19, 21, 58). Moreover, the varying risk of GBS observed for COVID-19 vaccines cannot be explained by the difference in the prefusion stability of full-length spike protein encoded by adenoviral, and mRNA vaccines, and present in subunit protein vaccines. Although ChAdOx1 adenovirus in AZD1222 encodes wild-type viral spike, which can lead to the sporadic presentation of post-fusion spike conformation on the cell surface (62), Ad26.COV2.S vaccine utilizes modified human adenovirus encoding stabilized prefusion spike protein (by substituting two residues with proline), similar to the spike encoded by both mRNA vaccines and present in NVX-CoV2373 subunit vaccine (63–66).

In summary, the evidence indicates that the increased risk of GBS following adenoviral vector vaccines against COVID-19 is more likely related to the response to administrated adenoviruses than the effect of cross-reactivity of anti-spike antibodies induced following immunization. This tentatively supports the hypothesis presented in this paper and suggests focusing further on selected adenoviral vectors as potential etiological agents of GBS.

### 3.2 Natural adenoviral infections and Guillain-Barré syndrome

Pathogen infections, including the gram-negative bacteria *Campylobacter jejuni* (32% of cases) and *Mycoplasma pneumoniae* (5%), and DNA viruses human cytomegalovirus (10-15%) and Epstein-Barr virus (8-10%), were implicated as one of the precipitants of GBS (67, 68). Such association with adenoviruses, potentially providing additional clues in understanding the increased risk of GBS after adenoviral vector vaccines, was substantially less explored. This is likely because adenoviral infections in immunocompetent individuals are mostly asymptomatic or self-limiting and do not require any specific treatment apart from supportive care (69). The first study addressing the potential association between adenoviral infections and GBS that included 92 GBS cases was published in 1977 and did not find such a relationship; instead, it highlighted that cytomegalovirus might be a common agent involved in the pathogenesis of GBS (70). The second study was published 25 years ago, analyzed 154 GBS cases, and reported the anti-adenovirus antibodies in 1% of samples while highlighting the association between antecedent *C*. *jejuni* infection and GBS and antiganglioside antibodies (68). However, the employed determinations in these studies were based on complement fixation, an old method, later suppressed by more accurate enzyme-linked immunosorbent assays (71, 72). Nevertheless, the findings of these studies likely caused no interest in further exploration of links between adenovirus infections and GBS using a more sensitive approach. The necessity to conduct such research reemergences now, after increased GBS risk was evidenced for selected adenoviral vector vaccines against COVID-19. Moreover, to evaluate the hypothesis put forward in the present paper, such investigations should distinguish between infections caused by different adenoviruses, with a primary focus on HAdV26, a component of Ad26.COV2.S vaccine, which administration was associated with an excess of GBS cases. In this context, the research should primarily focus on selected African and Asian populations since they demonstrate the highest seroprevalence of HAdV26 (73, 74). Although these regions are characterized by the lower reported GBS morbidity, it is acknowledged that this might be due to resource-limited settings resulting in a significant underestimation of cases (75). Including HAdV5 infections for comparison in such research would also be informative in understanding why no association between Ad5-nCoV vaccination and GBS was observed.

### 3.3 Adenovirus vaccination and Guillain-Barré syndrome

Most human adenoviruses are not associated with severe disease, and their infections are often asymptomatic (69). Therefore, there was no urgent need to develop, test and authorize the broadly available vaccine against adenoviruses. However, human adenovirus serotype 4 (HAdV4) and serotype 7 (HAdV7) have been reported to induce febrile acute respiratory disease and became a leading cause of hospitalization of U.S. Army personnel. To mitigate it, live oral vaccines have been used since the 1970s to protect U.S. soldiers from severe HAdV4 and HAdV7 infections (76, 77). The latest vaccine against these viruses, developed by Barr Labs, Inc., was licensed by the U.S. Food and Drug Administration in 2011 for military personnel aged 17-50. If exposure to adenoviral vectors was potentially a precipitant of GBS, one could expect to see cases of this condition following the administration of the adenovirus vaccine. In line with this, the analysis of the U.S. Vaccine Adverse Event Reporting System for reports among individuals who were immunized with the adenovirus vaccine between 2011 and 2018 found that GBS was the most frequently reported severe adverse events with the median onset time of 24 days from vaccination (78). However, one should note that the frequency of GBS was approximately 1.0 per 100,000 vaccinated individuals, which is within the background level (16). The authors speculate that these cases may also be due to the common co-administration of other vaccines and prophylactic intramuscular antibiotics in military personnel, complicating an understanding of the direct link with vaccination against adenoviruses (78). Nevertheless, such a link cannot be excluded and advocates further research, particularly in the light of GBS cases reported following adenoviral vector COVID-19 vaccines and the hypothesis outlined in the present paper.

### 3.4 Adenoviral vector vaccination and anti-vector antibodies

Administration of adenoviruses as vaccine vectors is associated with the induction of anti-vector antibodies, also in the case of modified non-replicating adenoviruses, which are classically obtained by deletion of E1/E3 region and lead to transient infection. As shown in phase 1 and 2/3 clinical trials of the AZD1222 vaccine, the administration of the first dose elicited anti-vector (anti-ChAdOx1) antibodies across different age groups, which remained detectable at high levels at least 84 days (last time point assessed) since vaccination (79, 80). ChAdOx1 is based on chimpanzee adenovirus Y25 with a very low baseline seroprevalence in the human population, which predisposed it as a good vector candidate (since pre-existing anti-vector immunity may decrease the efficacy of the vaccination). However, it was known that its broad use during the COVID-19 vaccination campaigns would increase the seroprevalence of anti-ChAdOx1 antibodies, while the potential cross-reactivity of these antibodies was not investigated.

Other authorized vector COVID-19 vaccines were based on human adenoviruses, HAdV5 and HAdV26 (**Table 2**). Administration of both of them should generate adaptive responses against them. For example, immunization with Ad26.COV2.S was associated with increased titers of antibodies neutralizing a HAdV26 that persisted for at least 71 days post-vaccination (last time point assessed) (88). According to the comprehensive international seroepidemiological study, the pre-pandemic seroprevalence of HAdV26 was significantly lower in all studied regions than that of HAdV5, for which it was widespread (73). This predisposed HAdV26 as a better candidate for the vaccine vector due to concern that pre-existing immunity to it could decrease the efficacy of immunization (89). However, further studies have shown that pre-existing immunity to a vector does not necessarily always prevent them from inducing a robust adaptive response to the target antigen, or if this is the case, such an effect can be overcome by increasing the dose of viral particles (90, 91). Pre-existing anti-HAdV26 antibodies did not compromise SARS-COV-2 neutralizing antibody responses to a booster (third) dose of the Sputnik V vaccine (based on the HAdV26 vector) (92). Moreover, as shown experimentally, anti-HAdV5 antibodies did not prevent HAdV5 from infecting muscle cells but contributed to the more rapid elimination of the vector, likely *via* effector mechanisms (93). Considering that the large epidemiological studies did not detect an increased risk of GBS following the administration of the Ad5-nCoV vaccine (25, 26) suggests that the pre-existing immunity to HAdV5 may play a protective role or that GBS risk is increased only during the first exposure to the virus, i.e., through natural infection. Contrary to HAdV5-based vaccines, a substantial number of individuals vaccinated with Ad26.CoV2.S did not have pre-existing immunity to a vector. Whether anti-HAdV26 antibodies may reveal cross-reaction with neuronal proteins associated with axonal and myelin function remains unclear.

**Table 2:** The overview of adenovirus vectors employed in COVID-19 vaccines.

Adenoviral vector	Code name	Group	NCBI taxonomy ID (txid)	Natural seroprevalence in human^1^	Replication competency of vector	Primary entry receptor ^2^
Human adenovirus type 5	HAdV5	C	28285	Very high: 60-70% in Europe, USA, >90% in Africa, Asia	incompetent	CAR
Human adenovirus type 26	HAdV26	D	46928	Moderate: 3-7% in Europe, 12-15% in the USA, 20-45% in Africa (over 60% in some regions, e.g., Botswana, Cameroon, Kenya, Uganda), 35-60% in Asia.	incompetent	sialic glycans
Chimpanzee adenovirus Y25	ChAdOx1	E	1123958	Very low: 0% in the UK, 9% in Gambia	incompetent	CAR

CAR, coxsackie and adenovirus receptor; ^1^, references (73, 74, 81–85); ^2^, references (86, 87).

Therefore, to address this knowledge gap and test the hypothesis outlined in the present paper, research based on a bioinformatic approach (e.g., through the construction of a protein-protein interaction network) and experimental studies (e.g., using purified anti-ChAdOx1, anti-HAdV26, and anti-HAdV5 antibodies and neuronal cell lines) are encouraged.

### 3.5 Alternative hypothesis: adenovirus infection of peripheral neurons

In addition to the above-discussed hypothesis on the role of anti-vector antibodies in GBS induction after administering adenoviral vector vaccines, this paper also offers an alternative hypothesis. According to it, the adenoviruses used in such vaccines can invade the peripheral nervous system, interact with a receptor on the surface of neurons, infect them, and trigger the immunological response that, sporadically, leads to GBS. Experimental *in vivo* studies have demonstrated that intramuscular injection of adenoviral vectors, including replication-deficient ones, can be transferred to the peripheral nervous system and deliver the genes of interest (94–96). As evidenced in animal studies, the administrated adenoviral vectors, including those which are replication-defective, can persist for weeks, not only locally but also in distant sites, e.g., the liver (97, 98). This makes their transfer to the peripheral nervous system more possible. In turn, as shown by the studies focusing on the central nervous system, the presence of adenovirus can stimulate T-cell responses leading to the elimination of the vector, but accompanied by inflammation (99, 100). What is important in light of the outlined hypothesis, this process can lead to local demyelination in the central nervous system (100–102). If this phenomenon is also plausible in the peripheral nervous system following intramuscular delivery of adenoviral vectors, it would explain the increased risk of GBS associated with administering selected adenoviral vector vaccines against COVID-19. As shown, the ChAdOx1 vector reveals a high affinity to the coxsackie and adenovirus receptor (CAR) (86), which is also expressed on the surface of neurons (103). Therefore, it is plausible that ChAdOx1 trafficking to the peripheral nervous system could, in some cases, induce the cascade leading to GBS. Whether the risk of such events is modified by the genetic predisposition postulated in GBS (28) remains to be studied. However, it has to be noted that HAdV5 also utilizes CAR as a primary cell receptor (104). Thus, the question arises as to why, contrary to AZD1222, the HAdV5-based Ad5-nCoV vaccination was not associated with excess GBS. One of the potential explanations that would require confirmatory studies is that pre-existing immunity to HAdV5 in vaccinated individuals, which had to be widespread, was efficient enough to suppress the translocation of vector to the peripheral nerves. The experimental observations support this scenario that increased baseline levels of anti-HadV5 antibodies contributed to more rapid elimination of the vector (93).

HAdV26 can also interact with CAR but reveals only a weak affinity. It has been previously suggested that this adenovirus utilizes the CD46 (105), which is also expressed on the surface of human neurons. More recent investigations have shown that CD46 is also unlikely a primary cell entry receptor for HAdV26 and established that this role is played by sialic acid–bearing glycans (87). Importantly, gangliosides, sialylated glycosphingolipids, are the most prevalent sialoglycans of nerve cells that reside primarily in the outer leaflet of the plasma membrane (106). Immune responses against gangliosides have been recognized to play a role in demyelinating immune-mediated neuropathies, including GBS (107). These observations tentatively favor the hypothesis outlined in this paper’s subsection. Whether HAdV26 can interact with gangliosides and induce such events remains to be studied *in vitro* and *in vivo*.

## 4 Conclusion

The risk of GBS following COVID-19 vaccination appears to be increased exclusively in the case of selected adenoviral vaccines, indicating that it is not the encoded antigen (spike protein) but a vector that is likely responsible for this neuropathy. This paper hypothesizes that some adenoviruses employed in vector vaccines can trigger an adaptive humoral immune response against themselves that, in some cases, can lead to the interaction of antibodies with neurological factors and induce GBS. Indeed, immunization with such vaccines is known to induce the production of anti-vector antibodies, which before the COVID-19 pandemic, were nearly non-existence in the case of anti-ChAdOx1 antibodies or had a relatively low prevalence for anti-HAdV26. In turn, the cross-reactivity of these antibodies was not studied.

According to the second hypothesis outlined in the present paper, the adenoviral particles in vector vaccines can, in some cases, invade the peripheral nervous system, interact with surface receptors (e.g., CAR), infect neurons, and induce an immune response that leads to GBS, the severity of which depends on the spectrum of neuronal inflammation. Such penetration and further consequences are plausible because the adenoviral vectors, including those devoid of replication potency, will persist at low levels for weeks after administration. Interestingly, GBS risk was not increased when using the COVID-19 vaccine based on the HAdV5 vector, for which the pre-existing immunity was widespread prior to the COVID-19 vaccination campaign. This suggests that using HAdV5 as a vaccine vector may potentially be beneficial in decreasing the vaccination-associated GBS risk due to pre-existing immunity to the vector and increased pace of vector elimination. However, this assumption would require further research confirmation.

Research testing the hypotheses mentioned above is urgently needed due to the ongoing interest in using adenoviruses in preventive vaccines against various infectious diseases and as immunotherapeutic agents in cancer treatment. Elucidating the underlying mechanism behind the GBS following adenoviral administration should be perceived as a pathway to increase the acceptance of vector vaccines because such knowledge will bring a better understanding of the risks and enable its elimination or precautionary actions.

## Data availability statement

The original contributions presented in the study are included in the article/supplementary material. Further inquiries can be directed to the corresponding author.

## Author contributions

PR conceptualized the study, outlined the hypotheses and wrote the manuscript.
